# Utility of SOFA score, management and outcomes of sepsis in Southeast Asia: a multinational multicenter prospective observational study

**DOI:** 10.1186/s40560-018-0279-7

**Published:** 2018-02-14

**Authors:** Khie Chen Lie, Chuen-Yen Lau, Nguyen Van Vinh Chau, T. Eoin West, Direk Limmathurotsakul, for Southeast Asia Infectious Disease Clinical Research Network, Pratiwi Sudarmono, Abu Tholib Aman, Mansyur Arif, Armaji Kamaludi Syarif, Herman Kosasih, Muhammad Karyana, Tawee Chotpitayasunondh, Warunee Punpanich Vandepitte, Adiratha Boonyasiri, Keswadee Lapphra, Kulkanya Chokephaibulkit, Pinyo Rattanaumpawan, Visanu Thamlikitkul, Achara Laongnualpanich, Prapit Teparrakkul, Pramot Srisamang, Phan Huu Phuc, Le Thanh Hai, Nguyen Van Kinh, Bui Duc Phu, Nguyen Thanh Hung, Tang Chi Thuong, Ha Manh Tuan, Lam Minh Yen, Nguyen Van Vinh Chau, Direk Limmathurotsakul, Janjira Thaipadungpanit, Stuart Blacksell, Nicholas Day, Claire Ling, Guy Thwaites, Heiman Wertheim, Le Van Tan, Motiur Rahman, H. Rogier van Doorn, Chuen-Yen Lau

**Affiliations:** 1 Department of Internal Medicine, Cipto Mangunkusumo Hospital, Jakarta, Indonesia; 2 Collaborative Clinical Research Branch, Division of Clinical Research, National Institute of Allergy and Infectious Diseases, National Institutes of Health, Bethesda, USA; 3 Department of Internal Medicine, Hospital for Tropical Diseases, Ho Chi Minh City, Vietnam; 4 Department of Internal Medicine, Oxford University Clinical Research Unit, Ho Chi Minh City, Vietnam; 5 Division of Pulmonary and Critical Care Medicine, Department of Medicine, University of Washington, Seattle, WA USA; 6 Department of Global Health, University of Washington, Seattle, WA USA; 7 Centre for Tropical Medicine and Global Health, Nuffield Department of Medicine, University of Oxford, Oxford, UK; 8 Mahidol-Oxford Tropical Medicine Research Unit, Faculty of Tropical Medicine, Mahidol University, Bangkok, Thailand; 9 Department of Tropical Hygiene, Faculty of Tropical Medicine, Mahidol University, 420/6 Rajvithi Road, Bangkok, 10400 Thailand

**Keywords:** Sepsis, Asia, Southeastern, Organ dysfunction scores, Patient care bundles

## Abstract

**Background:**

Sepsis is a global threat but insufficiently studied in Southeast Asia. The objective was to evaluate management, outcomes, adherence to sepsis bundles, and mortality prediction of maximum Sequential Organ Failure Assessment (SOFA) scores in patients with community-acquired sepsis in Southeast Asia.

**Methods:**

We prospectively recruited hospitalized adults within 24 h of admission with community-acquired infection at nine public hospitals in Indonesia (*n* = 3), Thailand (*n* = 3), and Vietnam (*n* = 3). In patients with organ dysfunction (total SOFA score ≥ 2), we analyzed sepsis management and outcomes and evaluated mortality prediction of the SOFA scores. Organ failure was defined as the maximum SOFA score ≥ 3 for an individual organ system.

**Results:**

From December 2013 to December 2015, 454 adult patients presenting with community-acquired sepsis due to diverse etiologies were enrolled. Compliance with sepsis bundles within 24 h of admission was low: broad-spectrum antibiotics in 76% (344/454), ≥ 1500 mL fluid in 50% of patients with hypotension or lactate ≥ 4 mmol/L (115/231), and adrenergic agents in 71% of patients with hypotension (135/191). Three hundred and fifty-five patients (78%) were managed outside of ICUs. Ninety-nine patients (22%) died. Total SOFA score on admission of those who subsequently died was significantly higher than that of those who survived (6.7 vs. 4.6, *p* < 0.001). The number of organ failures showed a significant correlation with 28-day mortality, which ranged from 7% in patients without any organ failure to 47% in those with failure of at least four organs (*p* < 0.001). The area under the receiver operating characteristic curve of the total SOFA score for discrimination of mortality was 0.68 (95% CI 0.62–0.74).

**Conclusions:**

Community-acquired sepsis in Southeast Asia due to a variety of pathogens is usually managed outside the ICU and with poor compliance to sepsis bundles. In this population, calculation of SOFA scores is feasible and SOFA scores are associated with mortality.

**Trial registration:**

ClinicalTrials.gov, NCT02157259. Registered 5 June 2014, retrospectively registered.

**Electronic supplementary material:**

The online version of this article (10.1186/s40560-018-0279-7) contains supplementary material, which is available to authorized users.

## Background

Sepsis, organ dysfunction due to a dysregulated host response to infection, is a major public health concern [1]. Sepsis is estimated to affect up to 20 million people around the world each year, and about 20–50% of people hospitalized with sepsis die [2]. Yet these estimates are extrapolations from high-income countries, home to only 13% of the world’s population. Sepsis is understudied in the low- and middle-income countries (LMICs) that host over six billion people [3].

Identification of sepsis—in the absence of a gold standard test—may be challenging [4, 5]. Recently, an international taskforce suggested that “sepsis” should be defined as life-threatening organ dysfunction caused by a dysregulated host response to infection and that the term “severe sepsis” was redundant [1]. The taskforce emphasized the use of the Sequential (sepsis-related) Organ Failure Assessment (SOFA) score [1], and organ dysfunction can be represented by an increase in the SOFA score of 2 points or more. Nonetheless, the SOFA score was derived in and has been primarily evaluated for mortality prediction in high-income countries [6–8]. There are few data about the mortality prediction of the SOFA score in LMICs and in non-ICU settings [9, 10].

Following diagnosis, successful sepsis management hinges on prompt treatment of infection and correction of organ dysfunction. Sepsis bundles such as those derived from Surviving Sepsis Campaign (SSC) guidelines facilitate management but have been primarily evaluated in high-income countries [11–15]. Relatively little is known about adherence to recommended sepsis bundles in LMICs. Healthcare systems in LMICs in Southeast Asia also vary. Thailand, an upper middle-income country, has a universal healthcare system with reasonably adequate coverage for the poor [16], while the healthcare systems in Vietnam and Indonesia, lower middle-income countries, still provide limited critical care coverage for patients with sepsis [17, 18]. Therefore, it is possible that management and outcomes of sepsis patients are different within LMICs in Southeast Asia.

We recently reported the causes and outcomes of 815 adult patients presenting with community-acquired infection in nine hospitals in three middle-income countries in Southeast Asia: Indonesia, Thailand, and Vietnam [19]. Sepsis was identified on enrolment in 454 adult patients and was associated with increased mortality. We observed that infection in this cohort was caused by a wide range of known and emerging pathogens, including dengue viruses, *Leptospira* spp., *Rickettsia* spp., *Escherichia coli*, and influenza viruses [19]. The hosts, infecting pathogens, and clinical capacity in this study are markedly different from those populations and sites evaluated in most studies of sepsis to date. However, a better understanding of sepsis management and outcomes in these environments is critically important to reduce the global burden of this syndrome. Here, we report the management and adherence to sepsis care bundles and mortality prediction of the SOFA score in adult patients with community-acquired sepsis in Southeast Asia.

## Methods

### Study sites and populations

We conducted a prospective cohort study of community-acquired sepsis and severe sepsis [20] in patients in nine public hospitals in Indonesia (*n* = 3), Thailand (*n* = 3), and Vietnam (*n* = 3) (Additional file 1: Figure S1). All are tertiary public hospitals equipped with microbiology facilities and ICUs, with a median bed number of 1000 (range 760–2200). Children and adults were enrolled in the study; the present analysis is limited to adults. The term “severe sepsis” in the previous study was based on diagnostic criteria from SSC 2012 [20] and was not used in this study in accordance with the most updated sepsis definition (sepsis-3) [1].

### Study participants

We prospectively enrolled adult patients (age ≥ 18 years) who were admitted with a primary diagnosis of suspected or documented infection made by the attending physician, were within 24 h of hospital admission, and had at least three of 20 modified SSC 2012 sepsis diagnostic criteria documented in the medical record (Additional file 2: Table S1) [19]. We excluded patients who were suspected of having hospital-acquired infections, had a hospital stay within 30 days prior to this admission, were transferred from other hospitals with a total duration of hospitalization > 72 h, or were enrolled in other clinical studies. For this analysis, we defined organ dysfunction as total SOFA score ≥ 2 and analyzed individuals meeting this criterion [1].

### Study procedures

The study was initiated in December 2013 in Thailand, March 2014 in Vietnam, and March 2015 in Indonesia and completed at all sites in December 2015. On enrollment, the study team used a standardized case report form (CRF) to record clinical symptoms and their respective durations, known chronic conditions, vital signs, Glasgow Coma Scale (GCS), fluid challenge (if performed), administration of oxygen and other drugs documented in the medical charts, results of laboratory tests performed by the study hospital laboratories, and primary diagnoses made by attending clinicians. Then, study nurses visited enrolled patients daily to update clinical information captured and to record final diagnoses made by attending clinicians at discharge.

Per protocol, the following rapid diagnostic tests (RDTs) were performed immediately after enrollment: a whole blood lactate RDT (Lactate Pro 2, Arkray Global Business Inc., Australia), a whole blood glucose RDT (ACCU-CHECK Performa, Roche Diagnostic, Germany), a dengue RDT (NS1 and IgM, Standard Diagnostics, South Korea), and a leptospirosis RDT (Leptospira IgM/IgG, Standard Diagnostics). The results of all rapid tests were reported to the attending physicians immediately. Blood samples were collected for culture on site and for serological tests and molecular tests at reference laboratory centers of each country. Other diagnostic specimens and a set of reference diagnostic tests were performed for each patient according to clinical presentation as previously described [19].

The study did not involve any clinical interventions. All treatment was provided by attending physicians and their medical teams. The 28-day mortality was evaluated via telephone contact if subjects were no longer hospitalized and had been discharged alive.

### Statistical analysis

Data were entered into OpenClinica, Enterprise Edition (Waltham, USA), and all analyses were performed using STATA version 14.0 (StataCorp, College Station, USA). This was a secondary analysis; the sample size of the study was determined for the primary objective of ascertaining etiology of sepsis [19].

Maximum SOFA scores within 24 h of admission for each of six organ systems were determined as shown in Additional file 3: Table S2. The cardiovascular SOFA score was modified slightly as the study protocol was not designed to capture the dosage of adrenergic agents in units of micrograms per kilogram per minute: one for mean arterial pressure < 70, two if dopamine was administered, and three if epinephrine or norepinephrine were administered. For a missing value, we used the closest available value from the pre-transfer period to 24 h of admission. Where no value was available, the predictor was assumed to be normal and given a score of 0. The total SOFA score was then calculated by summing the maximum SOFA scores for each of the six organ systems. For patients who required mechanical ventilation, the total GCS was estimated by the formula previously described [21]. Differences in proportions were evaluated using Fisher’s exact test and differences in medians by the Mann-Whitney test. The discriminative power of total SOFA score was defined by the area under the receiver operating characteristic curve (AUROC). We used logistic regression models stratified by study sites to evaluate the factors associated with mortality. Multivariable logistic regression models to evaluate the association between adherence to sepsis bundles and mortality were developed using purposeful selection [22] and were adjusted for age and total SOFA score within 24 h of admission.

## Results

**Fig. 1:**
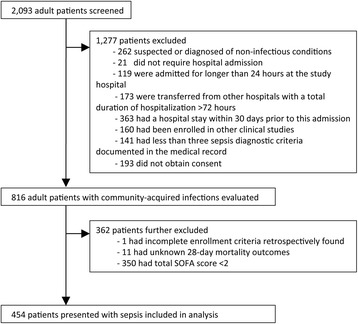
Study flow diagram. Some patients had more than one exclusion criteria

A total of 2093 adults presenting at nine study hospitals in three countries were screened by the study team (Fig. 1). The most common reasons for exclusion were hospitalization in the past 30 days (363, 14%) and suspicion or diagnosis of non-infectious conditions (262, 10%). Four hundred fifty-four adult patients had organ dysfunction as determined by total SOFA score ≥ 2. These patients were therefore deemed to have community-acquired sepsis and were included in the analysis (Table 1 and Additional file 4: Table S3). Of these, 219 patients (48%) were transferred from other hospitals.

**Table 1:** Baseline characteristics

Characteristics	No. of patients (%, *n* = 454)
Sex, male	287 (63%)
Age	
≥ 18–< 40 years	120 (26%)
≥ 40–< 60 years	169 (37%)
≥ 60 years old	165 (36%)
Country	
Indonesia	51 (11%)
Thailand	277 (61%)
Vietnam	126 (27%)
Preexisting known conditions	
Diabetes	88 (19%)
Hypertension	127 (28%)
Chronic kidney disease	45 (10%)
Chronic lung disease	21 (5%)
HIV/AIDS	0
Clinical presentations*	
Acute respiratory tract infection	243 (54%)
Acute diarrhea	107 (24%)
Acute central nervous system (CNS) infection	62 (14%)
Acute systematic infection	128 (28%)
SOFA score (mean, SD)^†^	5.0 ± 3.2

*The clinical presentations (in some cases, more than one) were defined based on the major presenting clinical symptoms. Acute respiratory tract infection was defined as manifestation of at least one respiratory symptom for no longer than 14 days. Acute diarrhea was defined as diarrhea for no longer than 14 days. Acute CNS infection was defined as manifestation of CNS symptoms for no longer than 14 days or the presence of signs of CNS infection on admission. Systemic infection was defined as the absence of acute respiratory infection, acute diarrhea, and acute CNS infection

^†^Total maximum SOFA scores from the pre-transfer period up to 24 h of admission

Four hundred forty-four patients (98%) had lactate levels measured on enrollment as part of the study protocol (Table 2). Of 231 patients presenting with sepsis-induced hypotension or lactate ≥ 4 mmol/L, 172 (74%) received an initial fluid challenge within 24 h after admission and 115 (50%) received ≥ 1500 mL during the fluid challenge. Of 77 patients who received ≥ 1500 mL during the fluid challenge and had body weight recorded, the median volume of fluid received was 42 mL/kg (IQR 34–56 mL/kg; range 19–122 mL/kg). Of 191 patients who had hypotension, 135 (71%) received an adrenergic agent, and norepinephrine was the most common adrenergic agent used (86%; 116/135) (Additional file 5: Table S4).

**Table 2:** Adherence to Surviving Sepsis Campaign care bundles up to 24 h after admission

Surviving Sepsis Campaign care bundles	Sepsis patients (*n* = 454)^†^
Measured lactate level	444 (98%)^‡^
Obtained blood culture	449 (99%)^‡^
Administered parenteral antibiotics	344 (76%)
Administered ≥ 1500 mL fluid for hypotension or lactate ≥ 4 mmol/L	115/231 (50%)
Administered adrenergic agent for hypotension	135/191 (71%)
Re-measured lactate level for hypotension or lactate ≥ 4 mmol/L	11/275 (4%)

Adapted from Rhodes et al. [23]

^†^Denominator is total *n* unless otherwise specified

^‡^Measuring lactate level and obtaining blood culture were part of the study protocol

Of 219 patients transferred from other hospitals, 137 (63%) had parenteral (intravenous or intramuscular) antibiotics administered prior to or during transfer. Another 207 patients had parenteral antibiotics administered at the study hospitals within 24 h of admission. Overall, the most common antibiotics used were ceftriaxone (71%; 245/344), ceftazidime (13%; 44/344), and carbapenems (11%; 37/344). Per study protocol, 449 patients (99%) had blood culture on enrollment. Reference diagnostic tests identified bacteria in 39% of patients (176/454), viruses in 16% (71/454), and parasites in 2% (9/454, Additional file 6: Table S5). *Leptospira* spp. (*n* = 52, 11%), dengue viruses (*n* = 46, 10%), *Escherichia coli* (*n* = 33, 7%), rickettsial pathogens (*n* = 18, 4%), *Streptococcus suis* (*n* = 14, 3%), and *Klebsiella pneumoniae* (*n* = 10, 2%) were the pathogens most commonly identified (Additional file 6: Table S5). Pathogens were not identified in 212 patients (47%).

Additional file 7: Table S6 shows other supportive care provided up to 24 h after admission. Seventy of 454 study patients (15%) were admitted directly to an ICU, and additional 29 patients (6%) were admitted to an ICU within 24 h after admission. Of 219 patients who were transferred from other hospitals, 154 (62%) had a peripheral oxygen saturation (SpO_2_) level documented at the outside facility, of whom 23 (15%) had a SpO_2_ < 90% (median 97%; IQR 93 to 99%; range 34 to 100%). On admission to the study hospital, 320 patients (70%) had a SpO_2_ level noted in the medical records, of whom 56 (18%) had a SpO_2_ < 90% (median 97%; IQR 92–99%; range 33 to 100%). Sixty-four patients (14%) were treated with mechanical ventilation. Per study protocol, 445 patients (98%) had whole blood glucose measured once on enrollment by the study team. Eight (2%) and 118 (26%) had severe hypoglycemia (blood glucose level < 40 mg/dL) and hyperglycemia (blood glucose level > 180 mg/dL), respectively.

**Fig. 2:**
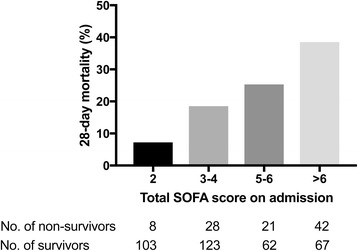
Twenty-eight-day mortality according to SOFA score up to 24 h of admission

The overall 28-day mortality was 22% (99/454). The 28-day mortality ranged from 7% (8/111) among those who had SOFA score = 2 to 39% among those who had SOFA score > 6 (Fig. 2). There were no clear differences for pathogens identified between survivors and non-survivors, except that *Leptospira* spp. and dengue viruses were more commonly identified in survivors (Additional file 8: Table S7). The mean total SOFA score was significantly higher in non-survivors than in survivors (6.7 vs. 4.6, OR 1.25; 95%CI 1.16–1.34, *p* < 0.001, Table 3). The odds of death increased with higher SOFA scores for all organ systems (*p* < 0.01 for all), except for the coagulation score (*p* = 0.89). The number of organ failures (where organ failure was defined as the maximum SOFA score ≥ 3 for an individual organ system) also showed a significant correlation with 28-day mortality, which ranged from 7% in patients without any organ failure to 47% in those with failure of at least four organs (*p* < 0.001; Additional file 9: Table S8). The AUROC of total SOFA score for discrimination of mortality was 0.68 (95% CI 0.62–0.74).

**Table 3:** Maximum SOFA scores up to 24 h of admission for the six organ systems in sepsis patients

System	Non-survivors* (*n* = 99)	Survivors* (*n* = 355)	Odds ratio	*p* values^†^
Respiration	1.2 (± 1.5)	0.3 (± 0.9)	1.70 (1.40–2.07)	< 0.001
Coagulation	1.1 (± 1.4)	1.4 (± 1.3)	1.01 (0.93–1.23)	0.89
Liver	1.0 (± 1.4)	0.6 (± 1.1)	1.34 (1.10–1.63)	0.004
Cardiovascular	1.2 (± 0.9)	0.8 (± 0.9)	1.80 (1.35–2.40)	< 0.001
Central nervous system	0.6 (± 0.6)	0.3 (± 0.7)	1.67 (1.24–2.25)	0.001
Renal	1.6 (± 1.3)	1.1 (± 1.3)	1.27 (1.06–1.52)	0.009
Total SOFA score	6.7 (± 3.8)	4.6 (± 2.9)	1.25 (1.16–1.34)	< 0.001

*Results are presented as mean (± standard deviation)

^†^*p* value estimated by univariable logistic regression stratified by study site

Determination of SOFA scores is based on both clinical and laboratory parameters [6, 7]. We found that some laboratory tests were not available or measured routinely for sepsis patients within 24 h after admission in our middle-income country settings. Most of the patients had both platelet (*n* = 452, 99%) and creatinine (*n* = 443, 98%) tests performed, while 65% (*n* = 294) and 25% (*n* = 113) had bilirubin and arterial blood gas tests performed, respectively (Additional file 10: Table S9). While every patient had vital signs documented, 64% (*n* = 291) had GCS values documented in the medical charts prior to the enrollment.

In logistic regression models adjusted for age and SOFA score and stratified by study site (Table 4), we found that adherence to sepsis care bundles within 24 h was not associated with survival. Using tests for interactions, we found that relationships between SOFA score and mortality and between sepsis management and mortality were not significantly different among subgroup of patients with viruses or bacteria identified.

**Table 4:** Factors associated with 28-day mortality in adult patients with sepsis

Factors	Outcome		Odds ratio (95% CI)*	
	Non-survivors (*n* = 99)^‡^	Survivors (*n* = 355)^‡^	Univariable analysis	Multivariable analysis^†^
Admitted directly to ICU	19 (19%)	51 (14%)	3.8 (1.8–8.1, *p* < 0.001)	1.9 (0.8–4.5, *p* = 0.16)
Administered parenteral antibiotics	87 (88%)	257 (72%)	3.4 (1.6–7.0, *p* = 0.001)	1.7 (0.7–3.9, *p* = 0.22)
Administered ≥ 1500 mL fluid for hypotension or lactate ≥ 4 mmol/L	32/72 (44%)	83/159 (52%)	0.9 (0.5–1.7, *p* = 0.73)	0.8 (0.4–1.5, *p* = 0.43)
Administered adrenergic agent for hypotension	40/52 (77%)	95/139 (68%)	1.7 (0.8–3.6, *p* = 0.20)	1.4 (0.6–3.1, *p* = 0.45)

*Stratified by study sites

^†^Adjusted for age and total SOFA score

^‡^Denominator is total *n* unless otherwise specified

## Discussion

This prospective observational study characterized management and outcomes of patients with diverse etiologies of community-acquired sepsis in three middle-income countries in Southeast Asia. The main findings from this study are that adherence to SSC bundles even at the late time point of 24 h after admission is generally low. Most patients are not admitted to the ICU but are managed on the wards. The 28-day mortality is 22%. Despite incomplete laboratory and certain clinical data, calculation of SOFA scores with minor modifications is feasible and the total SOFA score within 24 h of admission is strongly correlated with mortality.

Sepsis care bundles have been developed to facilitate the implementation of core tenets of sepsis treatment guidelines [11, 23]. In middle-income countries, where resources are relatively limited compared to high-income countries, it is important to determine how sepsis care is provided as the applicability of or ability to implement these guidelines may be impaired [24, 25]. Our study only permitted assessment of adherence to bundles at 24 h, yet even at this late time point, sepsis care bundles were applied to patients presenting with community-acquired sepsis at variable rates. The high adherence of measuring lactates (98%) and obtaining blood culture (99%) was largely driven by the study protocol and may not reflect the current standard of care for community-acquired sepsis in Southeast Asia. Timely antimicrobial therapy is considered an essential component of sepsis treatment [20], but only 76% of patients received a parenteral antibiotic within the first 24 h. Notably, viruses, parasites, spirochetes, and rickettsial pathogens were identified in 33% of patients with sepsis. It is possible, for example, that if clinicians suspect or confirm by rapid diagnostic tests that the sepsis is caused by a virus such as dengue virus, antimicrobial therapies may not be administered [26]. However, distinguishing these patients based on clinical presentation is challenging and may result in inadequate treatment of bacterial infection. Intravenous fluid resuscitation in this study was generally restrictive.

Optimal fluid management of patients with sepsis in high-resource settings remains debated [27]. Intravenous fluid resuscitation in this study was generally restrictive. Of particular concern is the risk-benefit profile of fluid administration in low-resource settings [28, 29]. Despite the potential mortality benefits of following sepsis bundles, compliance has historically been low in many settings [23]. Proactive strategies for increasing compliance are necessary and may be uniquely challenging in limited resource settings. Approaches that may have benefit include educational campaigns, engaging a full-time intensivist, establishing nurse-driven protocols, and providing feedback to clinicians regarding specific performance metrics [14, 15]. Together, our findings underscore the importance of evaluating and prioritizing fundamental elements of sepsis care in Southeast Asia with an emphasis on efficacy, cost-effectiveness, and feasibility.

We found that performance of components of sepsis bundles within 24 h of admission was not associated with survival outcomes. It is possible that those therapies were not provided within 3 and 6 h, preferred benchmarks for sepsis treatment [23]; therefore, their benefits were not observed. The lack of benefit of administration of parenteral antibiotics and fluid challenge could also be due to residual confounding factors. Unfortunately, the information documented in the medical record in our settings was not adequate to estimate whether the bundles were performed within 3 or 6 h of admissions. While these findings should not be interpreted as indicating negative or no impact of sepsis care bundles in sepsis patients, they highlight the importance of understanding etiologies and evaluating management strategies for sepsis in different clinical environments.

SOFA scores permit the determination of organ dysfunction, using a combination of clinical and laboratory variables [6]. We found that, for the most part, SOFA scores can be measured with the available standard of care resources in middle-income countries in Southeast Asia. Nearly every patient presenting with sepsis had blood collected for platelet count and creatinine level on admission, whereas about half had bilirubin levels measured. Therefore, the additional cost for laboratory tests would be either minimal or moderate for middle-income countries. Cardiovascular and central nervous system scores—determined from clinical parameters—could be readily measured without additional cost. Although our study did not capture doses of adrenergic agents—necessitating a modification of the cardiovascular SOFA score—this information is nonetheless available in clinical practice. However, the SOFA respiration score is based on the PaO_2_ from an arterial blood gas (to calculate PaO_2_/FiO_2_). We observed that only 25% of our sepsis patients had arterial blood gas testing performed, perhaps reflecting the added complexity of arterial blood sampling or need for specialized testing equipment. This issue no doubt underestimated the severity of illness in many cases. For example, patients who required mechanical ventilation due to sepsis-associated hypoxemic respiratory failure, yet did not have an arterial blood gas drawn, received a respiratory SOFA score of 0. To overcome these challenges, reframing the SOFA respiratory score for LMICs by using SpO_2_/FiO_2_ [30] or simply based on a requirement for supplemental oxygen or respiratory support devices may be helpful.

In our study cohort, despite a weak AUROC for discrimination of 28-day mortality, total SOFA score was nonetheless robustly associated with 28-day mortality. This association was also valid for individual organ SOFA scores, with the exception of the coagulation score. This may be a spurious finding or due to the wide range of pathogens responsible for sepsis in our study. Previous studies evaluating the relationship of SOFA score with mortality have been performed in locations where most sepsis is attributed to bacterial infections [6, 9, 31]. Thrombocytopenia in those who have bacterial infections may be caused by disseminated intravascular coagulation and thus is highly associated with mortality [6, 9, 31]. It is possible that thrombocytopenia in those with viral infections, such as dengue, is not so strongly associated with mortality as that observed in those with bacterial infections. Thrombocytopenia is also common in leptospirosis patients [32], and overall mortality of leptospirosis (about 7% in the recent review [33] and 8% [4/52] in our study) is generally lower than in sepsis patients without *Leptospira* spp. identified (24% [95/402] in our study). Therefore, the limited predictability of thrombocytopenia observed in our study was possibly because dengue and leptospirosis were common, and mortality in patients in whom these pathogens were identified was lower than in patients with other pathogens (Additional file 8: Table S7). Tests for interaction [22] and our sample size may lack power to evaluate this phenomenon. Nonetheless, we raise the concern that the SOFA coagulation score for sepsis in tropical countries in Southeast Asia—where causes of infections are diverse [19, 34]—may not provide a linear contribution to the prediction of mortality as has been observed in other settings.

Our study has several potential limitations. First, the actual standard of care could be higher than what we observed due to lack of documentation in the medical records or lower due to observational bias. Second, we may have excluded some patients with organ dysfunction and the true SOFA score could be higher if arterial blood gas levels, bilirubin levels, dose of adrenergic agents in units of micrograms per kilogram per minute, and GCS were measured and documented in all patients; nonetheless, our results represent the real situation in middle-income countries in Southeast Asia.

## Conclusions

Our study characterizes the management and outcomes of sepsis due to a diversity of pathogens in public hospitals in Southeast Asia. We identify areas for improvement in sepsis care and show that the SOFA score is generally feasible to quantify the degree of organ dysfunction and determine the risk of death in these patients. To reduce mortality caused by sepsis in LMICs, the fundamental elements of sepsis care need to be tailored to and evaluated in these settings.

## Additional files

Additional file 1: **Figure S1.** Study sites. Red dots represent nine study areas. (1) Jakarta, (2) Yogyakarta and (3) Makassar in Indonesia; (4) Bangkok, (5) Chiang Rai and (6) Ubon Ratchathani in Thailand; and (7) Hanoi, (8) Hue and (9) Ho Chi Minh City in Vietnam. (TIFF 9143 kb)

Additional file 2: **Table S1.** Diagnostic criteria for sepsis in adult patients. (DOCX 63 kb)

Additional file 3: **Table S2.** Sequential (sepsis-related) Organ Failure Assessment Score. (DOCX 64 kb)

Additional file 4: **Table S3.** Baseline characteristics and mortality by country. (DOCX 65 kb)

Additional file 5: **Table S4.** Adherence to Surviving Sepsis Campaign care bundles up to 24 h after admission by country. (DOCX 62 kb)

Additional file 6: **Table S5.** Pathogens identified by country. (DOCX 72 kb)

Additional file 7: **Table S6.** Other supportive care provided from the pre-transfer period up to 24 h after admission by country. (DOCX 63 kb)

Additional file 8: **Table S7.** Pathogens identified in non-survivors and survivors. (DOCX 71 kb)

Additional file 9: **Table S8.** Number of organ system failures (maximum SOFA score ≥ 3 points) up to 24 h of admission and 28-day mortality in sepsis patients. (DOCX 61 kb)

Additional file 10: **Table S9.** Availability of tests to calculate SOFA scores up to 24 h of admission by country. (DOCX 61 kb)
