# Supplementary figures and images for: Utility of SOFA score, management and outcomes of sepsis in Southeast Asia: a multinational multicenter prospective observational study
Source: J Intensive Care. 2018 Feb 14;6:9. doi: 10.1186/s40560-018-0279-7 (PMC5813360; doi:10.1186/s40560-018-0279-7)

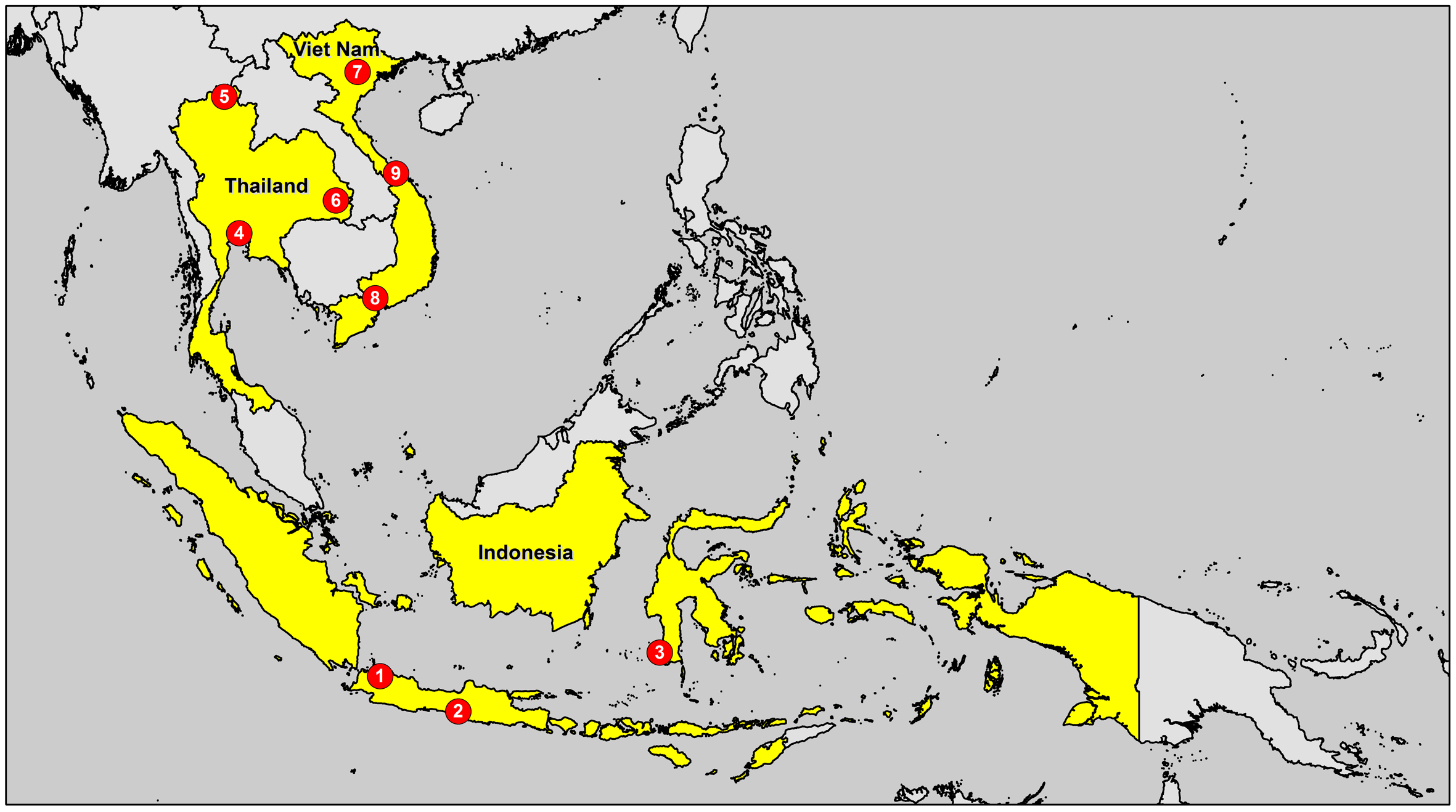

Supplement: Supplementary file 1 — Figure S1. Study sites. Red dots represent nine study areas. (1) Jakarta, (2) Yogyakarta and (3) Makassar in Indonesia; (4) Bangkok, (5) Chiang Rai and (6) Ubon Ratchathani in Thailand; and (7) Hanoi, (8) Hue and (9) Ho Chi Minh City in Vietnam. (TIFF 9143 kb) [file 40560_2018_279_MOESM1_ESM.tif]
