# Supplementary material for: Utility of SOFA score, management and outcomes of sepsis in Southeast Asia: a multinational multicenter prospective observational study
Source: J Intensive Care. 2018 Feb 14;6:9. doi: 10.1186/s40560-018-0279-7 (PMC5813360; doi:10.1186/s40560-018-0279-7)
Supplement: Supplementary file 2 — Table S1. Diagnostic criteria for sepsis in adult patients. (DOCX 63 kb) [file 40560_2018_279_MOESM2_ESM.docx]

**Table S1: Diagnostic criteria for sepsis in adult patients**

| **Infection, documented or suspected, and some of the following:** |
| --- |
| **General variables** |
| Fever or hypothermia (body temperature >38.3^o^C or <36^o^C) * |
| Heart rate >90/min |
| Tachypnoea (respiratory rate >20/min) |
| Altered mental status (Glasgow Coma Scale <15 or <10T) † |
| Significant oedema or positive fluid balance (20 mL/kg over 24 hr) |
| Hyperglycaemia (plasma glucose >140 mg/dL) in the absence of diabetes |
| **Inflammatory variables** |
| Leukocytosis (WBC count >12,000/μL), Leukopenia (WBC count <4,000/μL), or immature forms >10% ‡ |
| Plasma C-reactive protein more than two SD above the normal value |
| Plasma procalcitonin more than two SD above the normal value |
| **Haemodynamic variables** |
| Arterial hypotension (SBP <90 mmHg, MAP <70 mmHg or an SBP decrease >40 mmHg) |
| **Organ dysfunction variables** |
| Low oxygen saturation determined by pulse oximetry (SpO_2_ <95%) § |
| Arterial hypoxaemia (PaO_2_/FiO_2_ <300) |
| Acute oliguria (urine output <0.5 mL/kg/hr for at least 2 hrs) ¶ |
| Creatinine increase >0.5 mg/dL |
| Coagulation abnormalities (INR >1.5 or aPTT >60s) |
| Ileus (absent bowel sounds) |
| Thrombocytopenia (platelet count <100,000/μL) |
| Hyperbilirubinaemia (plasma total bilirubin >4 mg/dL) |
| **Tissue perfusion variables** |
| Hyperlactataemia (>1 mmol/L) |
| Decreased capillary refill or mottling |

Adapted from Dellinger et al. [20]

* Variables fever and hypothermia were consolidated into a single variable.

† Glasgow Coma Scale <15 or <10T was defined for the altered mental status variable.

‡ Variables leukocytosis, leukopenia and immature forms >10% were consolidated into a single variable.

§ Variable low oxygen saturation determined by pulse oximetry (SpO_2_ <95%) was added.

¶ There is a condition of ‘despite adequate fluid resuscitation’ for this criterion in the SSC 2012 diagnostic criteria for severe sepsis.
