# Supplementary material for: Utility of SOFA score, management and outcomes of sepsis in Southeast Asia: a multinational multicenter prospective observational study
Source: J Intensive Care. 2018 Feb 14;6:9. doi: 10.1186/s40560-018-0279-7 (PMC5813360; doi:10.1186/s40560-018-0279-7)
Supplement: Supplementary file 3 — Table S2. Sequential (sepsis-related) Organ Failure Assessment Score. (DOCX 64 kb) [file 40560_2018_279_MOESM3_ESM.docx]

**Table S2. Sequential (Sepsis-Related) Organ Failure Assessment Score**

|  | Score |  |  |  |  |
| --- | --- | --- | --- | --- | --- |
| System | 0 | 1 | 2 | 3 | 4 |
| Respiration |  |  |  |  |  |
| PaO_2_/FiO_2_, mmHg | ≥400 | <400 | <300 | <200 with respiratory support | <100 with respiratory support |
| Coagulation |  |  |  |  |  |
| Platelets, /μL | ≥150,000 | <150,000 | <100,000 | <50,000 | <20,000 |
| Liver |  |  |  |  |  |
| Bilirubin, mg/dl | <1.2 | 1.2-1.9 | 2.0-5.9 | 6.0-11.9 | >12.0 |
| Cardiovascular |  |  |  |  |  |
| MAP and  adrenergic agents | MAP≥70 mmHg | MAP<70mmHg | Dopamine or dobutamine (any dose) | Epinephrine  or norepinephrine  (any dose) | - |
| Central nervous system |  |  |  |  |  |
| Glasgow Coma Scale  score | 15 | 13-14 | 10-12 | 6-9 | <6 |
| Renal |  |  |  |  |  |
| Creatinine, mg/dL | <1.2 | 1.2-1.9 | 2.0-3.4 | 3.5-4.9 | >5.0 |
| Urine output, mL/d |  |  |  | <500 | <200 |

Adapted from Singer et al. [1]. For the cardiovascular component of the SOFA score, the scoring was modified such that subjects were scored a maximum of 2 (on a 4- point scale) if they received only dobutamine or dopamine, and scored a maximum of 3 if they received epinephrine or norepinephrine.

Abbreviations: FiO2, fraction of inspired oxygen, MAP, mean arterial pressure, PaO2, partial pressure oxygen
