# Supplementary material for: Utility of SOFA score, management and outcomes of sepsis in Southeast Asia: a multinational multicenter prospective observational study
Source: J Intensive Care. 2018 Feb 14;6:9. doi: 10.1186/s40560-018-0279-7 (PMC5813360; doi:10.1186/s40560-018-0279-7)
Supplement: Supplementary file 4 — Table S3. Baseline characteristics and mortality by country. (DOCX 65 kb) [file 40560_2018_279_MOESM4_ESM.docx]

**Table S3. Baseline characteristics and mortality by country**

| **Characteristics** | **Indonesia**  **(%, n=51)** | **Thailand**  **(%, n=277)** | **Viet Nam**  **(%, n=126)** |
| --- | --- | --- | --- |
| **Sex, male** | 29 (57%) | 175 (63%) | 83 (66%) |
| **Age** |  |  |  |
| ≥18 - <40 years | 13 (26%) | 57 (21%) | 50 (40%) |
| ≥40 - <60 years | 19 (37%) | 93 (33%) | 57 (45%) |
| ≥60 years old | 19 (37%) | 127 (46%) | 19 (15%) |
| **Preexisting known conditions** |  |  |  |
| Diabetes | 10 (20%) | 63 (23%) | 15 (12%) |
| Hypertension | 14 (27%) | 98 (35%) | 15 (12%) |
| Chronic kidney disease | 5 (10%) | 36 (13%) | 4 (3%) |
| Chronic lung disease | 9 (18%) | 10 (4%) | 2 (2%) |
| HIV/AIDS | 0 | 0 | 0 |
| **Clinical presentations *** |  |  |  |
| Acute respiratory tract infection | 29 (57%) | 160 (58%) | 54 (43%) |
| Acute diarrhoea | 7 (14%) | 83 (30%) | 17 (13%) |
| Acute central nervous system (CNS) infection | 2 (4%) | 49 (18%) | 11 (8%) |
| Acute systematic infection | 16 (31%) | 59 (21%) | 53 (42%) |
| **SOFA score (mean, SD)** † | 4.6 ± 2.5 | 5.5 ± 3.3 | 4.2 ± 3.2 |

* The clinical presentations (in some cases, more than one) were defined based on the major presenting clinical symptoms. Acute respiratory tract infection was defined as manifestation of at least one respiratory symptom for no longer than 14 days. Acute diarrhoea was defined as diarrhoea for no longer than 14 days. Acute CNS infection was defined as manifestation of CNS symptoms for no longer than 14 days or presence of signs of CNS infection on admission. Systemic infection was defined as absence of acute respiratory infection, acute diarrhoea and acute CNS infection.

† Total maximum SOFA scores from the pre-transfer period up to 24 hours of admission
