# Supplementary material for: Utility of SOFA score, management and outcomes of sepsis in Southeast Asia: a multinational multicenter prospective observational study
Source: J Intensive Care. 2018 Feb 14;6:9. doi: 10.1186/s40560-018-0279-7 (PMC5813360; doi:10.1186/s40560-018-0279-7)
Supplement: Supplementary file 5 — Table S4. Adherence to Surviving Sepsis Campaign care bundles up to 24 h after admission by country. (DOCX 62 kb) [file 40560_2018_279_MOESM5_ESM.docx]

**Table S4. Adherence to Surviving Sepsis Campaign care bundles up to 24 hours after admission by country ***

| **Surviving Sepsis Campaign care bundles** | **Indonesia**  **(%, n=51)** † | **Thailand**  **(%, n=277)** † | **Viet Nam**  **(%, n=126)** † |
| --- | --- | --- | --- |
| Measured lactate level | 44 (86%) | 277 (100%) | 123 (98%) |
| Obtained blood culture | 47 (92%) | 277 (100%) | 125 (99%) |
| Administered parenteral antibiotics | 37 (73%) | 224 (81%) | 83 (66%) |
| Administered ≥1,500 mL fluid for hypotension or lactate ≥4mmol/L | 2/32 (6%) | 101/176 (57%) | 12/23 (52%) |
| Administered adrenergic agent for hypotension | 7/14 (50%) | 117/161 (73%) | 11/16 (69%) |
| Re-measured lactate level for hypotension or lactate ≥4mmol/L | 0/32 (0%) | 6/176 (4%) | 5/23 (22%) |

* Adapted from Rhodes et al. [23]

† Denominator is total n unless otherwise specified

‡ Measuring lactate level and obtaining blood culture were part of the study protocol
