# Supplementary material for: Utility of SOFA score, management and outcomes of sepsis in Southeast Asia: a multinational multicenter prospective observational study
Source: J Intensive Care. 2018 Feb 14;6:9. doi: 10.1186/s40560-018-0279-7 (PMC5813360; doi:10.1186/s40560-018-0279-7)
Supplement: Supplementary file 6 — Table S5. Pathogens identified by country. (DOCX 72 kb) [file 40560_2018_279_MOESM6_ESM.docx]

**Table S5. Pathogens identified by country**

| **Pathogens identified** | **Indonesia** | **Thailand** | **Vietnam** |
| --- | --- | --- | --- |
| **Bacteria** |  |  |  |
| **Gram-negative bacteria** |  |  |  |
| **Salmonella enterica** |  |  |  |
| *S. enterica* serovar Typhi | 2 (3.9%) | 0 (0.0%) | 1 (0.8%) |
| Non-typhi *Salmonella* | 0 (0.0%) | 8 (2.9%) | 0 (0.0%) |
| **Non-salmonella enterobacteriaceae** |  |  |  |
| *Escherichia coli* | 3 (5.9%) | 23 (8.3%) | 7 (5.6%) |
| *Klebsiella pneumoniae* | 0 (0.0%) | 6 (2.2%) | 4 (3.2%) |
| *Klebsiella* spp | 0 (0.0%) | 3 (1.1%) | 0 (0.0%) |
| *Enterobacter* spp | 1 (2.0%) | 4 (1.4%) | 1 (0.8%) |
| *Citrobacter* spp | 0 (0.0%) | 2 (0.7%) | 0 (0.0%) |
| *Shigella* spp | 0 (0.0%) | 0 (0.0%) | 0 (0.0%) |
| **Other Gram-negative** |  |  |  |
| *Acinetobacter* spp | 1 (2.0%) | 3 (1.1%) | 0 (0.0%) |
| *Burkholderia pseudomallei* | 0 (0.0%) | 3 (1.1%) | 0 (0.0%) |
| *Haemophilus* spp | 0 (0.0%) | 0 (0.0%) | 0 (0.0%) |
| *Pseudomonas* spp | 0 (0.0%) | 0 (0.0%) | 0 (0.0%) |
| *Campylobacter* spp | 0 (0.0%) | 0 (0.0%) | 0 (0.0%) |
| *Chlamydophila pneumoniae* | 0 (0.0%) | 0 (0.0%) | 0 (0.0%) |
| *Bordetella* spp | 0 (0.0%) | 0 (0.0%) | 0 (0.0%) |
| *Acromobacter* spp | 0 (0.0%) | 0 (0.0%) | 0 (0.0%) |
| *Aeromonas* spp | 0 (0.0%) | 0 (0.0%) | 0 (0.0%) |
| *Legionella* spp | 0 (0.0%) | 0 (0.0%) | 0 (0.0%) |
| *Vibrio* spp | 0 (0.0%) | 1 (0.4%) | 0 (0.0%) |
| Unspecified Gram-negative | 0 (0.0%) | 2 (0.7%) | 0 (0.0%) |
| **Gram-positive bacteria** |  |  |  |
| *Staphylococcus aureus* | 0 (0.0%) | 5 (1.8%) | 1 (0.8%) |
| *Streptococcus pneumoniae* | 1 (2.0%) | 2 (0.7%) | 2 (1.6%) |
| *Streptococcus suis* | 0 (0.0%) | 6 (2.2%) | 8 (6.3%) |
| Beta-hemolytic *Streptococcus* spp | 0 (0.0%) | 8 (2.9%) | 0 (0.0%) |
| Unspecified Gram-positive | 0 (0.0%) | 0 (0.0%) | 0 (0.0%) |
| **Other bacteria** |  |  |  |
| *Leptospira* spp | 4 (7.8%) | 35 (12.6%) | 13 (10.3%) |
| Rickettsial pathogens |  |  |  |
| *Orientia tsutsugamushi* | 1 (2.0%) | 13 (4.7%) | 4 (3.2%) |
| *Rickettsia* *typhi* | 1 (2.0%) | 2 (0.7%) | 2 (1.6%) |
| Spotted fever group rickettsia | 0 (0.0%) | 6 (2.2%) | 5 (4.0%) |
| *Mycoplasma* spp | 0 (0.0%) | 0 (0.0%) | 0 (0.0%) |
| *Mycobacterium tuberculosis* | 0 (0.0%) | 0 (0.0%) | 1 (0.8%) |
| **Virus** |  |  |  |
| Dengue virus | 3 (5.9%) | 21 (7.6%) | 22 (17.5%) |
| Influenza | 2 (3.9%) | 4 (1.4%) | 2 (1.6%) |
| Hantavirus | 0 (0.0%) | 1 (0.4%) | 5 (4.0%) |
| Rotavirus | 1 (2.0%) | 1 (0.4%) | 0 (0.0%) |
| Norovirus | 1 (2.0%) | 0 (0.0%) | 0 (0.0%) |
| Cytomegalovirus | 1 (2.0%) | 0 (0.0%) | 0 (0.0%) |
| Japanese encephalitis virus | 0 (0.0%) | 0 (0.0%) | 0 (0.0%) |
| Herpes Simplex virus | 0 (0.0%) | 1 (0.4%) | 0 (0.0%) |
| Epstein-Barr virus | 0 (0.0%) | 1 (0.4%) | 0 (0.0%) |
| Rhinovirus | 0 (0.0%) | 4 (1.4%) | 1 (0.8%) |
| Respiratory syncytial virus | 0 (0.0%) | 2 (0.7%) | 0 (0.0%) |
| Adenovirus | 0 (0.0%) | 0 (0.0%) | 0 (0.0%) |
| Parainfuenza virus | 0 (0.0%) | 1 (0.4%) | 0 (0.0%) |
| Bocarvirus | 0 (0.0%) | 0 (0.0%) | 0 (0.0%) |
| Enterovirus | 0 (0.0%) | 1 (0.4%) | 0 (0.0%) |
| Metapneumovirus | 0 (0.0%) | 1 (0.4%) | 0 (0.0%) |
| Coronarivus | 1 (2.0%) | 0 (0.0%) | 0 (0.0%) |
| Parechovirus | 0 (0.0%) | 0 (0.0%) | 0 (0.0%) |
| **Fungus** |  |  |  |
| *Candida* spp | 0 (0.0%) | 1 (0.4%) | 0 (0.0%) |
| **Parasite** |  |  |  |
| *Plasmodium* spp | 0 (0.0%) | 3 (1.1%) | 3 (2.4%) |
| *Entamoeba histolytica* | 0 (0.0%) | 1 (0.4%) | 0 (0.0%) |
| *Stronglyloides stercoralis* | 0 (0.0%) | 2 (0.7%) | 0 (0.0%) |
| *Cryptosporidium* spp | 0 (0.0%) | 0 (0.0%) | 0 (0.0%) |
| **Total number of pathogens identified** |  |  |  |
| 0 | 31 (60.8%) | 127 (45.8%) | 53 (42.1%) |
| 1 | 17 (33.3%) | 130 (46.9%) | 65 (51.6%) |
| ≥2 | 3 (5.9%) | 20 (7.2%) | 8 (6.3%) |
| **Total number of patients** | 51 (100.0%) | 277 (100.0%) | 126 (100.0%) |
