# Supplementary material for: Utility of SOFA score, management and outcomes of sepsis in Southeast Asia: a multinational multicenter prospective observational study
Source: J Intensive Care. 2018 Feb 14;6:9. doi: 10.1186/s40560-018-0279-7 (PMC5813360; doi:10.1186/s40560-018-0279-7)
Supplement: Supplementary file 7 — Table S6. Other supportive care provided from the pre-transfer period up to 24 h after admission by country. (DOCX 63 kb) [file 40560_2018_279_MOESM7_ESM.docx]

**Table S6. Other supportive care provided from the pre-transfer period up to 24 hours after admission by country**

| **Supportive care *** | **Indonesia**  **(%, n=51)** | **Thailand**  **(%, n=277)** | **Viet Nam**  **(%, n=126)** |
| --- | --- | --- | --- |
| Admission directly to intensive care units | 1 (2%) | 22 (8%) | 47 (37%) |
| Measured peripheral capillary oxygen saturation | 27 (53%) | 264 (95%) | 51 (40%) |
| Measured blood gas level | 23 (45%) | 56 (20%) | 34 (27%) |
| Required mechanical ventilation | 3 (6%) | 55 (20%) | 6 (5%) |
| Deep vein thrombosis prophylaxis | 2 (4%) | 4 (1%) | 1 (1%) |
| Stress ulcer prophylaxis | 35 (69%) | 114 (41%) | 33 (26%) |
| Received renal replacement therapy |  |  |  |
| Peritoneal dialysis | 0 | 2 (1%) | 0 |
| Hemodialysis | 1 (2%) | 4 (1%) | 1 (1%) |
| Imaging performed |  |  |  |
| Chest radiography | 20 (39%) | 237 (86%) | 80 (63%) |
| Ultrasonography | 7 (14%) | 21 (8%) | 56 (44%) |
| CT scan | 9 (18%) | 26 (9%) | 0 |
| MRI | 0 | 1 (0.4%) | 0 |

* Adapted from Rhodes et al. [23]
