# Supplementary material for: Utility of SOFA score, management and outcomes of sepsis in Southeast Asia: a multinational multicenter prospective observational study
Source: J Intensive Care. 2018 Feb 14;6:9. doi: 10.1186/s40560-018-0279-7 (PMC5813360; doi:10.1186/s40560-018-0279-7)
Supplement: Supplementary file 8 — Table S7. Pathogens identified in non-survivors and survivors. (DOCX 71 kb) [file 40560_2018_279_MOESM8_ESM.docx]

**Table S7. Pathogens identified in non-survivors and survivors**

| **Pathogens identified** | **Non-survivors (n=99)** | **Survivors**  **(n=355)** | **P values** |
| --- | --- | --- | --- |
| **Bacteria** |  |  |  |
| **Gram-negative bacteria** |  |  |  |
| **Salmonella enterica** |  |  |  |
| *S. enterica* serovar Typhi | 0 (0.0%) | 3 (0.8%) | >0.99 |
| Non-typhi *Salmonella* | 0 (0.0%) | 8 (2.3%) | 0.21 |
| **Non-salmonella enterobacteriaceae** |  |  |  |
| *Escherichia coli* | 7 (7.1%) | 26 (7.3%) | >0.99 |
| *Klebsiella pneumoniae* | 2 (2.0%) | 8 (2.3%) | >0.99 |
| *Klebsiella* spp | 0 (0.0%) | 3 (0.8%) | >0.99 |
| *Enterobacter* spp | 2 (2.0%) | 4 (1.1%) | 0.62 |
| *Citrobacter* spp | 0 (0.0%) | 2 (0.6%) | >0.99 |
| *Shigella* spp | 0 (0.0%) | 0 (0.0%) | N/A |
| **Other Gram-negative** |  |  |  |
| *Acinetobacter* spp | 1 (1.0%) | 3 (0.8%) | >0.99 |
| *Burkholderia pseudomallei* | 2 (2.0%) | 1 (0.3%) | 0.12 |
| *Haemophilus* spp | 0 (0.0%) | 0 (0.0%) | N/A |
| *Pseudomonas* spp | 0 (0.0%) | 0 (0.0%) | N/A |
| *Campylobacter* spp | 0 (0.0%) | 0 (0.0%) | N/A |
| *Chlamydophila pneumoniae* | 0 (0.0%) | 0 (0.0%) | N/A |
| *Bordetella* spp | 0 (0.0%) | 0 (0.0%) | N/A |
| *Acromobacter* spp | 0 (0.0%) | 0 (0.0%) | N/A |
| *Aeromonas* spp | 0 (0.0%) | 0 (0.0%) | N/A |
| *Legionella* spp | 0 (0.0%) | 0 (0.0%) | N/A |
| *Vibrio* spp | 0 (0.0%) | 1 (0.3%) | >0.99 |
| Unspecified Gram-negative | 2 (2.0%) | 0 (0.0%) | 0.05 |
| **Gram-positive bacteria** |  |  |  |
| *Staphylococcus aureus* | 3 (3.0%) | 3 (0.8%) | 0.12 |
| *Streptococcus pneumoniae* | 2 (2.0%) | 3 (0.8%) | 0.30 |
| *Streptococcus suis* | 3 (3.0%) | 11 (3.1%) | >0.99 |
| Beta-hemolytic *Streptococcus* spp | 3 (3.0%) | 5 (1.4%) | 0.38 |
| Unspecified Gram-positive | 0 (0.0%) | 0 (0.0%) | N/A |
| **Other bacteria** |  |  |  |
| *Leptospira* spp | 4 (4.0%) | 48 (13.5%) | 0.007 |
| Rickettsial pathogens |  |  |  |
| *Orientia tsutsugamushi* | 1 (1.0%) | 17 (4.8%) | 0.14 |
| *Rickettsia* *typhi* | 2 (2.0%) | 3 (0.8%) | 0.30 |
| Spotted fever group rickettsia | 2 (2.0%) | 9 (2.5%) | >0.99 |
| *Mycoplasma* spp | 0 (0.0%) | 0 (0.0%) | N/A |
| *Mycobacterium tuberculosis* | 0 (0.0%) | 1 (0.3%) | >0.99 |
| **Virus** |  |  |  |
| Dengue virus | 3 (3.0%) | 43 (12.1%) | 0.007 |
| Influenza | 2 (2.0%) | 6 (1.7%) | 0.69 |
| Hantavirus | 0 (0.0%) | 6 (1.7%) | 0.35 |
| Rotavirus | 0 (0.0%) | 2 (0.6%) | >0.99 |
| Norovirus | 0 (0.0%) | 1 (0.3%) | >0.99 |
| Cytomegalovirus | 1 (1.0%) | 0 (0.0%) | 0.22 |
| Japanese encephalitis virus | 0 (0.0%) | 0 (0.0%) | N/A |
| Herpes Simplex virus | 0 (0.0%) | 1 (0.3%) | >0.99 |
| Epstein-Barr virus | 0 (0.0%) | 1 (0.3%) | >0.99 |
| Rhinovirus | 2 (2.0%) | 3 (0.8%) | 0.30 |
| Respiratory syncytial virus | 0 (0.0%) | 2 (0.6%) | >0.99 |
| Adenovirus | 0 (0.0%) | 0 (0.0%) | N/A |
| Parainfuenza virus | 0 (0.0%) | 1 (0.3%) | >0.99 |
| Bocarvirus | 0 (0.0%) | 0 (0.0%) | N/A |
| Enterovirus | 0 (0.0%) | 1 (0.3%) | >0.99 |
| Metapneumovirus | 0 (0.0%) | 1 (0.3%) | >0.99 |
| Coronarivus | 0 (0.0%) | 1 (0.3%) | >0.99 |
| Parechovirus | 0 (0.0%) | 0 (0.0%) | N/A |
| **Fungus** |  |  |  |
| *Candida* spp | 1 (1.0%) | 0 (0.0%) | 0.22 |
| **Parasite** |  |  |  |
| *Plasmodium* spp | 0 (0.0%) | 6 (1.7%) | 0.35 |
| *Entamoeba histolytica* | 0 (0.0%) | 1 (0.3%) | >0.99 |
| *Stronglyloides stercoralis* | 0 (0.0%) | 2 (0.6%) | >0.99 |
| *Cryptosporidium* spp | 0 (0.0%) | 0 (0.0%) | N/A |
| **Total number of pathogens identified** |  |  |  |
| 0 | 58 (58.6%) | 153 (43.1%) |  |
| 1 | 37 (37.4%) | 175 (49.3%) |  |
| ≥2 | 4 (4.0%) | 27 (7.6%) |  |
| **Total number of patients** | 99 (100.0%) | 355 (100.0%) |  |
