# Supplementary material for: Utility of SOFA score, management and outcomes of sepsis in Southeast Asia: a multinational multicenter prospective observational study
Source: J Intensive Care. 2018 Feb 14;6:9. doi: 10.1186/s40560-018-0279-7 (PMC5813360; doi:10.1186/s40560-018-0279-7)
Supplement: Supplementary file 9 — Table S8. Number of organ system failures (maximum SOFA score ≥ 3 points) up to 24 h of admission and 28-day mortality in sepsis patients. (DOCX 61 kb) [file 40560_2018_279_MOESM9_ESM.docx]

**Table S8. Number of organ system failures (maximum** **SOFA score** ≥**3 points) up to 24 hours of admission and 28-day mortality in sepsis patients**

| Number of organ system failures | 28-day mortality | Total SOFA score * |
| --- | --- | --- |
| 0 | 7% (8/111) | 2.0 (± 0.0) † |
| 1 | 27% (41/154) | 4.1 (± 1.2) |
| 2 | 22% (29/133) | 6.0 (± 2.2) |
| 3 | 34% (14/41) | 10.4 (± 2.3) |
| ≥4 | 47% (7/15) | 13.6 (± 2.1) |

* Results are presented as mean (± standard deviation)

† All 111 patients had Total SOFA score of 2
