# Supplementary material for: Utility of SOFA score, management and outcomes of sepsis in Southeast Asia: a multinational multicenter prospective observational study
Source: J Intensive Care. 2018 Feb 14;6:9. doi: 10.1186/s40560-018-0279-7 (PMC5813360; doi:10.1186/s40560-018-0279-7)
Supplement: Supplementary file 10 — Table S9. Availability of tests to calculate SOFA scores up to 24 h of admission by country. (DOCX 61 kb) [file 40560_2018_279_MOESM10_ESM.docx]

**Table S9. Availability of tests to calculate SOFA scores up to 24 hours of admission by country**

| **Organ System and Tests** | **Indonesia**  **(%, n=51)** | **Thailand**  **(%, n=277)** | **Viet Nam**  **(%, n=126)** |
| --- | --- | --- | --- |
| Respiration: Arterial blood gas for PaO_2_ | 23 (45%) | 56 (20%) | 34 (27%) |
| Coagulation: Platelets | 51 (100%) | 276 (99%) | 125 (99%) |
| Liver: Bilirubin | 10 (20%) | 229 (83%) | 55 (44%) |
| Cardiovascular: Blood pressure | 51 (100%) | 277 (100%) | 126 (100%) |
| Central Nervous System: Glasgow Coma Scale | 50 (98%) | 225 (81%) | 16 (13%) |
| Renal: Creatinine | 48 (94%) | 273 (99%) | 48 (94%) |
